# Small mammal glucocorticoid concentrations vary with forest fragment size, trap type, and mammal taxa in the Interior Atlantic Forest

**DOI:** 10.1038/s41598-021-81073-2

**Published:** 2021-02-04

**Authors:** Sarah A. Boyle, Noé U. de la Sancha, Pastor Pérez, David Kabelik

**Affiliations:** 1 Department of Biology and Program in Environmental Studies and Sciences, Rhodes College, Memphis, TN USA; 2 Department of Biological Sciences, Chicago State University, Chicago, IL USA; 3 The Field Museum, Integrative Research Center, Chicago, IL USA; 4 Facultad Politécnica, Universidad Nacional de Asunción, Asunción, Paraguay; 5 Department of Biology and Program in Neuroscience, Rhodes College, Memphis, TN USA

**Keywords:** Conservation biology, Ecophysiology, Tropical ecology

## Abstract

Species that live in degraded habitats often show signs of physiological stress. Glucocorticoid hormones (e.g., corticosterone and cortisol) are often assessed as a proxy of the extent of physiological stress an animal has experienced. Our goal was to quantify glucocorticoids in free-ranging small mammals in fragments of Interior Atlantic Forest. We extracted glucocorticoids from fur samples of 106 small mammals (rodent genera *Akodon* and *Oligoryzomys*, and marsupial genera *Gracilinanus* and *Marmosa*) from six forest fragments (2–1200 ha) in the Reserva Natural Tapytá, Caazapá Department, Paraguay. To our knowledge, this is the first publication of corticosterone and cortisol levels for three of the four sampled genera (*Akodon*, *Oligoryzomys*, and *Marmosa*) in this forest system. We discovered three notable results. First, as predicted, glucocorticoid levels were higher in individuals living withing small forest fragments. Second, animals captured live using restraint trapping methods (Sherman traps) had higher glucocorticoid levels than those animals captured using kill traps (Victor traps), suggesting that hair glucocorticoid measures can reflect acute stress levels in addition to long-term glucocorticoid incorporation. These acute levels are likely due to urinary steroids diffusing into the hair shaft. This finding raises a concern about the use of certain trapping techniques in association with fur hormone analysis. Finally, as expected, we also detected genus-specific differences in glucocorticoid levels, as well as cortisol/corticosterone ratios.

## Introduction

Habitat loss and fragmentation are primary threats to biodiversity^1,2^. It is estimated that at least 75% of Earth’s non-ice terrestrial surfaces have been modified by humans^3^. Forest loss and fragmentation can impact climate and microclimate, as well as species distributions, ecology, and behavior^4–6^. However, species responses to habitat loss and fragmentation vary^7,8^.

Habitat loss and fragmentation are also associated with stress, immunosuppression, and disease in wildlife^9,10^, which can directly impact populations^11^. Habitat fragmentation has been linked to population declines as a consequence of prolonged chronic stress^12,13^, and such stress can lead to potentially deleterious behavioral and physiological conditions^13^. However, higher levels of glucocorticoids (energy-mobilizing hormones whose circulating concentrations are often raised during times of stress) are not always clearly attributed to animals in forest fragments^14,15^, suggesting that physiological responses are complex and varied^16–18^. Thus, understanding how organisms physiologically respond to changes in their habitats is extremely valuable^19^.

Glucocorticoids are steroid hormones, whose blood concentrations tend to follow a circadian rhythm related to activity level, as well as rising during times of stress so as to help mobilize stored nutrients in order to enable an individual to deal with the stressor^20^. While cortisol is the dominant glucocorticoid secreted by most mammals, some mammal species primarily secrete corticosterone^21^. Because glucocorticoids are released in a pulsatile manner across minutes, and because they exhibit daily and event-related fluctuations, blood sampling provides an instantaneous snapshot of blood concentration, which may not be reflective of long-term levels. Because steroid hormones and their metabolites are continually deposited within fur, sampling of steroid levels within fur can provide longer-term quantification of mean hormone levels over periods of days to months^21–24^, which is a more appropriate time-scale when quantifying glucocorticoid levels for individual animals that may be in traps for more than several hours.

Typically, the increase in the intensity of the stressor correlates with glucocorticoid increases^20^, and chronic stress can negatively impact fitness through the inhibition of reproduction^20,25^. However, some individuals are capable of several physiological responses that may ultimately block the additional secretion of stress-related glucocorticoids or physiologically compensate for the increased levels, while others are able to maintain reproductive behavior during stressful times^25^. Experimental manipulation of glucocorticoids in wild animals also result in inconsistent effects on physiology, behavior, and fitness; these differences in responses could be due to differences in species, sex, age class, environmental conditions, and methodologies^26^. Furthermore, the relationship between baseline glucocorticoid levels and fitness can vary, even within populations and individuals^17^. However, the connections between stress and population dynamics in many species have highlighted the conservation implications of long-term stress on individuals^27,28^. Understanding the glucocorticoid response of a variety of organisms (from the individual to species level) is important for gaining insight on the effects of environmental changes on the stress response of various organisms^16^.

In mammals, higher glucocorticoids have been associated with social stress, dominance rank, reproductive status, disease, resource availability, season, age, sex of the individual, environmental change, and human disturbance^29–34^. However, specific studies examining the stress response of animals in the context of habitat fragmentation, habitat degradation, and human influences have found varying results. For example, multiple studies found higher levels of glucocorticoids in habitats experiencing greater modifications and pressures by humans^35–40^, but other studies found no differences in glucocorticoids between animals in forest fragments and continuous forest^14^ or between animals in national parks and suburban backyards^41^. Although there have been numerous studies on the impacts of stress in small mammals in laboratories^42,43^, there have been few studies published on glucocorticoid levels in small mammals in the wild^44,45^, and overall information on the endocrinology of many taxa is limited^46^.

The Interior Atlantic Forest of Paraguay, located in the eastern half of the country, has undergone recent and dramatic change: Most of this forested area was intact as recently as the 1970s, but by 2003 only 13.4% of the original Interior Atlantic Forest remained^47^. Forest loss has continued^48^, and it is likely that this ecosystem will be impacted by climate change^49^. Furthermore, the mammals of Paraguay remain among the least-studied in South America^50,51^, with major knowledge gaps present in most groups^50,52^.

The purpose of our work was to determine how glucocorticoid concentrations varied in small mammals in a highly disturbed forest system. Specifically, we tested to what extent variables associated with the small mammals (e.g., genus, species, and ecomorphological factors such as arboreal vs. cursorial), sampling methods (e.g., trap type, if the animal was dead or alive immediately prior to fur sampling, and trap placement on the ground, in a pitfall, or elevated), and the size of the forest fragment correlated with glucocorticoid levels. Because glucocorticoid levels have been shown to vary across taxa^53^, we predicted there would be differences in small mammal genera and species. Based on previous findings that found a relationship between the extent of habitat disturbance and glucocorticoid levels^36,39,40^, we predicted that glucocorticoid levels would negatively correlate with the size of the forest fragment in which the animals were captured, so that small mammals in smaller forest fragments would have higher glucocorticoid levels than small mammals in larger forest fragments. However, because patterns have varied in studies of animals in human-impacted landscapes, and there can be confounding factors that affect glucocorticoid levels^53^, including the impact of capture stress^54^, we included ecomorphological factors and sampling methods in the analyses. Given that factors associated with glucocorticoid levels are not fully apparent broadly across taxa, as well as within small mammal taxa, our research findings can contribute information about the physiological ecology of wild small mammals living in fragmented landscapes.

## Results

### Individuals sampled

The 106 individuals sampled across the six forest fragments represented five species of rodents (*Akodon montensis*, *A*. *paranaensis*, *Oligoryzomys mattogrossae*, *O*. *flavescence*, and *O*. *nigripes*) and two species of marsupials (*Gracilinanus agilis*, *Marmosa paraguayana*) in four genera. *Oligoryzomys* and *Akodon* were the most common (56.6% and 34.9% of the captured individuals, respectively; Supplementary Fig. 1), and *O. nigripes* represented 93.3% of its genus and *A*. *montensis* represented 62.2% of its genus. For *Akodon*, 40.5% were female (n = 15), 54.1% were male (n = 20), and 5.4% were of unknown sex (n = 2). For *Oligoryzomys*, 46.7% were female (n = 28), 50.0% were male (n = 30), and 3.3% were of unknown sex (n = 2). All individuals of unknown sex appeared to be adults, based on body mass. For *G. agilis*, 71.4% were female (n = 5), and 28.6% were male (n = 2). For *M. paraguayana*, 50.0% were female (n = 1), 50.0% were male (n = 1). Individuals were primarily trapped on the ground (84.0%, n = 89, including pitfall captures), followed by trapped in trees approximately 1.5 m from the ground (16.0%, n = 17), but the percentage of captures on the ground ranged from 100.0% for *Akodon* to 83.3% for *Oligoryzomys* to 28.6% for *G. agilis* to 0.0% for *M. paraguayana*. Overall, 50.0% of the individuals were captured with Sherman traps, indicating that 50.0% of the animals were in their trap alive prior to sampling of the fur. The remaining half of the individuals were captured via large Victor snap traps (31.1% of the individuals), followed by pitfall traps and small Victor snap traps (11.3% and 7.5% of the individuals, respectively).

### Glucocortocoid discriminant function analyses

**Figure 1:**
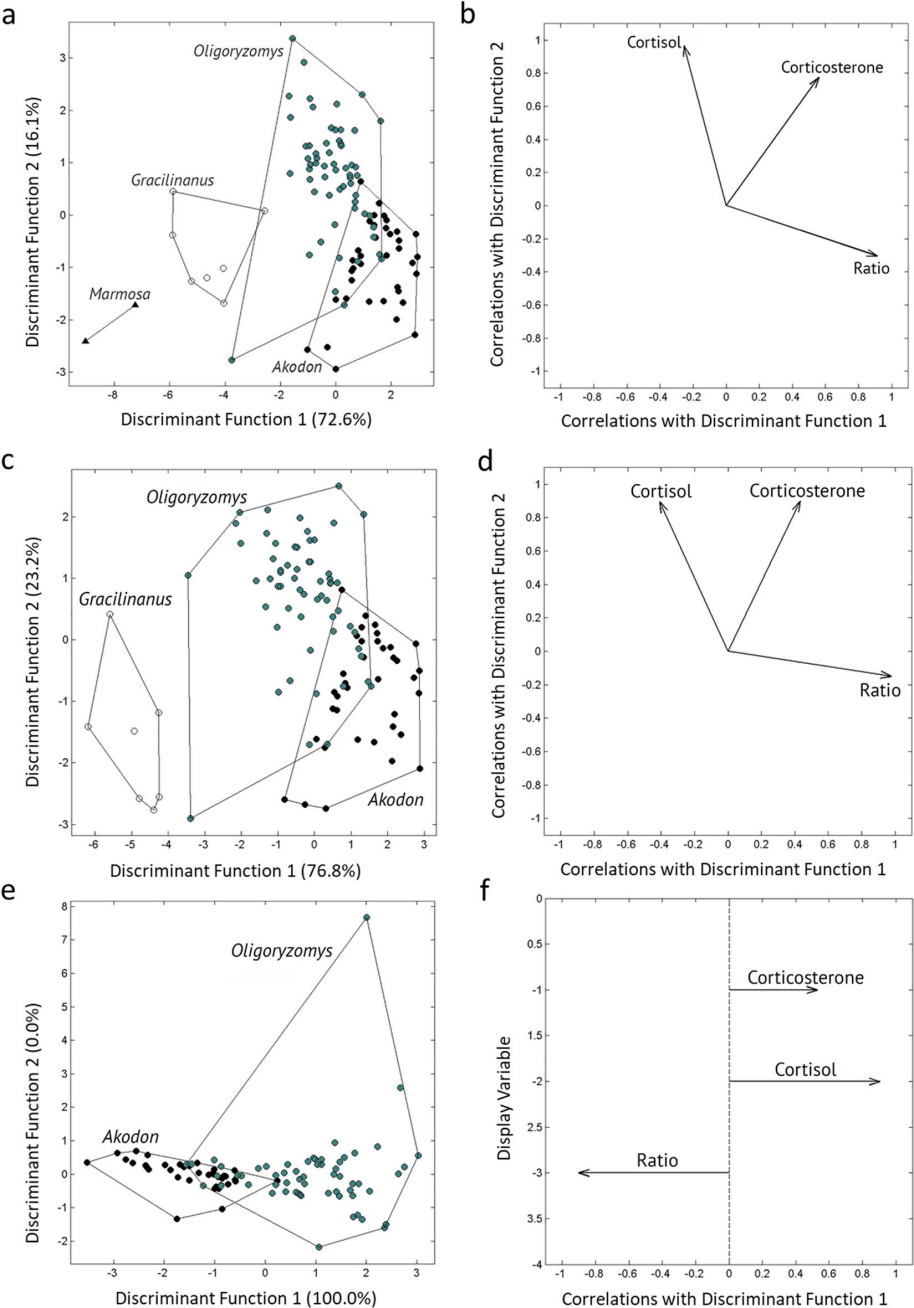
Discriminant function analysis (DFA) results for log-transformed hormone levels (corticosterone, cortisol, and ratio of the two hormones) for the two rodent genera (*Akodon* and *Oligoryzomys*) and the two marsupial genera (*Marmosa* and *Gracilinanus*) via (**a**) scatterplots and (**b**) biplots; DFA results between the two rodent genera (*Akodon* and *Oligoryzomys*) and the marsupial *Gracilinanus* via (**c**) scatterplots and (**d**) biplots; and DFA results between the two rodent genera (*Akodon* and *Oligoryzomys*) via (**e**) scatterplots and (**f**) biplots in forest fragments of the Atlantic Forest of eastern Paraguay. The axes for the scatterplots (**a**,**c**,**e**) represent the projection of the first two dimensions of the discriminant function analyses; the percentages noted on the axes represent the variation between groups per dimension. The biplots (**b**,**d**,**f**) show the eigenvalues for each of the variables that were used to discriminate between genera. The y-axis for the rodent biplot (**f**) is a dummy variable for display only because there is only one discrimination dimension.

Glucocorticoid levels were significantly different among the four genera of small mammals (Wilks’ λ = 0.0792, F_9,243.5_ = 49.6, p < 0.0001), but glucocorticoids were more similar between the two rodent genera than between rodents and marsupials (Table 1; Fig. 1a,b). When we compared the two rodent genera and the marsupial *Gracilinanus* (excluding *Marmosa* due to low sample size), we found glucocorticoid levels were significantly different among these three genera (Wilks’ λ = 0.1652, F_6,198_ = 48.2, p < 0.0001), and the marsupial (genus *Gracilinanus*) samples were fully separated from the rodent genera in their glucocorticoid levels (Fig. 1c,d). Finally, when we compared only the two rodent genera, we found that glucocorticoid levels were significantly different between *Akodon* and *Oligoryzomys* (Wilks’ λ = 0.4226, F_1,93_ = 42.3, p < 0.0001), although some overlap occurred between individuals representing the two rodent genera (Fig. 1e,f).

**Table 1:** Small mammal corticosterone and cortisol concentrations for *Oligoryzomys*, *Akodon*, *Gracilinanus* and *Marmosa*.

	*O. mattagrossae*	*O. flavescence*	*O. nigripes*	*A. montensis*	*A. paranaensis*	*G. agilis*	*M. paraguayana*
Samples (N)	2	2	56	23	14	7	2
**Corticosterone**							
Mean ± SE (pg/mg)	1403.3 ± 689.1	498.7 ± 106.6	986.2 ± 95.3	454.4 ± 66.1	479.6 ± 68.6	42.3 ± 13.6	23.5 ± 7.5
**Cortisol**							
Mean ± SE (pg/mg)	248.6 ± 198.0	53.2 ± 5.0	209.1 ± 34.0	24.7 ± 4.5	21.01 ± 2.8	75.3 ± 28.5	34.6 ± 5.3
**Corticosterone + cortisol**							
Mean ± SE (pg/mg)	1651.9 ± 887.1	551.9 ± 111.6	1195.3 ± 112.0	479.1 ± 70.1	500.6 ± 70.4	117.6 ± 41.3	58.1 ± 12.7
**Corticosterone-cortisol ratio**							
Mean ± SE	9.4 ± 4.7	9.3 ± 1.1	7.5 ± 0.6	20.8 ± 2.2	24.4 ± 2.9	0.7 ± 0.1	0.7 ± 0.1

### Glucocorticoid generalized linear models

**Figure 2:**
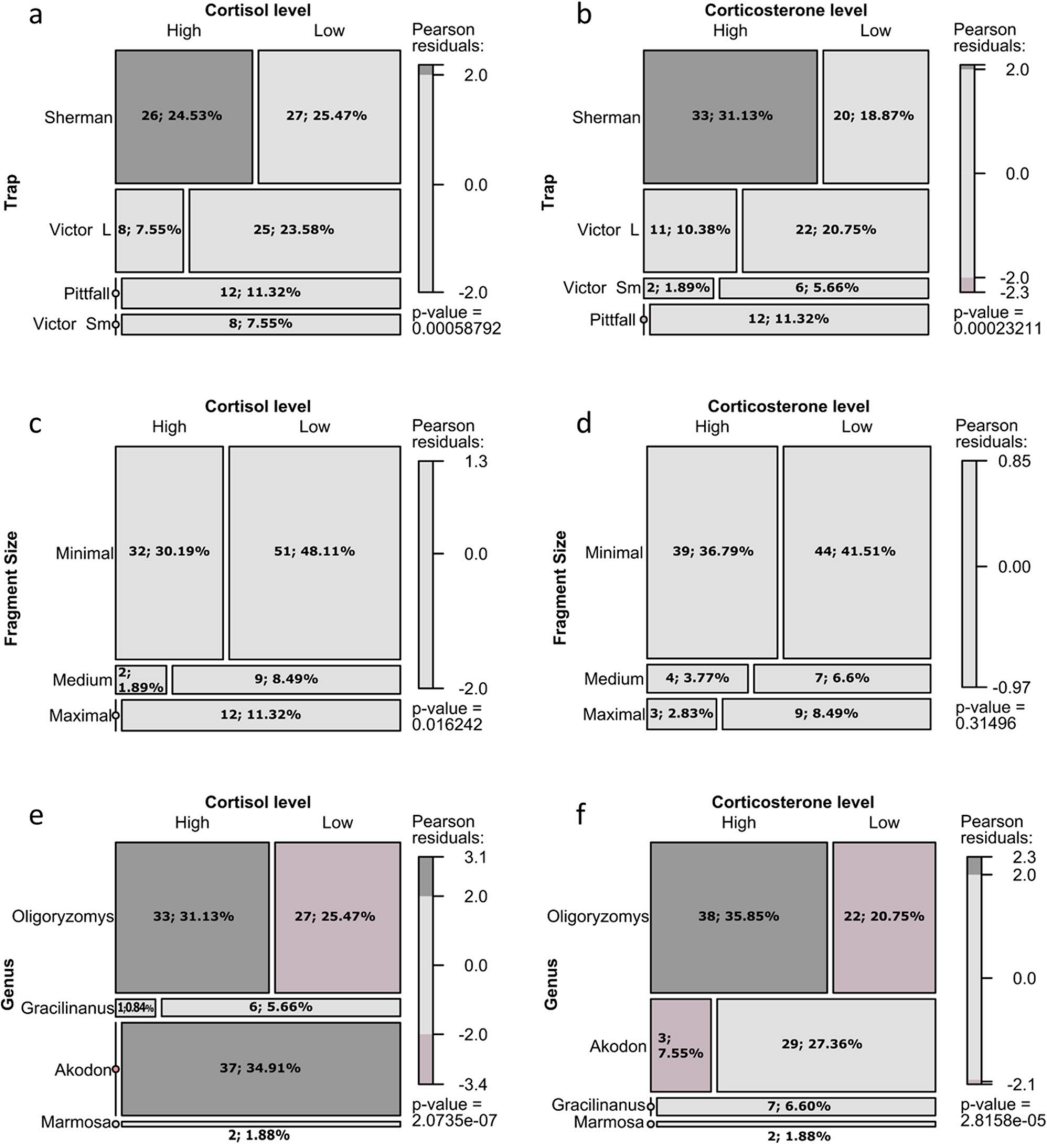
MosaicPlot of levels of cortisol and corticosterone (low and high) given the area (large, medium, and small) of forest fragments (**a**,**b**), type of trap (**c**,**d**), and genus (**e**,**f**). The size of the cell depends on the frequencies observed in the cross-classification of the contingency tables, and cells without numbers have a frequency of zero. With each plot is a Pearson residue reference scale. The sign of the residual indicates whether the observed frequency in a cell is greater or less than the expected value, and the magnitude indicates the degree of deviation from expected.

Logistic models indicated that forest fragment area, mammal taxon, and capture mode (type of trap) were related to glucocorticoid levels; this hypothesis was statistical supported for corticosterone (Table 2) and cortisol (Table 3) independently. The model with the lowest value of AICc (the best model) for corticosterone was the model with Area, Genus, and Trap included as fixed predictor variables with an additive effect [f(Area + Genus + Trap)]; the same finding occurred for cortisol (Tables 2 and 3 provide a comparison of all models). The performance of the best model by the area under the receiver operating characteristics curve (AUC) indicated a good test for corticosterone [0.75, 0.9) and a very good test [0.9, 0.97) for cortisol. Low levels of glucocorticoids were more frequent in large areas, and the frequency of high levels of the two hormones evaluated increased for medium and smaller areas (cortisol: G = 11.3, df = 2, p = 0.003, Fig. 2a; and corticosterone: G = 2.3, df = 2, p = 0.3, Fig. 2b). We also found that the type of trap was associated with glucocorticoid levels: lower levels of glucocorticoids were more frequent with pitfall and small Victor traps, followed by large Victor traps, and the frequency of high levels of glucocorticoids increased with the use of Sherman traps (cortisol: G = 21.9, df = 3, p < 0.0001, Fig. 2c; and corticosterone: G = 22.8, df = 3, p < 0.0001, Fig. 2d). Note that this pattern corresponded to live- and dead-capture traps. In addition, comparisons across the four genera found that *Akodon* showed a greater frequency of low levels of stress hormones (27.3% for corticosterone and 34.9% for cortisol), as did the two genera of marsupials; in contrast, *Oligoryzomys* had an increase in the frequency (31.1% for corticosterone and 35.8% for cortisol) of high levels of glucocorticoids (cortisol: G = 39.4, df = 3, p < 0.0001, Fig. 2e; and corticosterone: G = 24.7, df = 3, p < 0.0001, Fig. 2f). In summary, the size of the forest fragment, taxonomic group (i.e., rodent, marsupial), and method of capture were components that explained glucocorticoid levels.

**Table 2:** Comparison of generalized linear models used to describe corticosterone levels in all small mammal species in forest fragment samples.

Corticosterone logistic models	Rank	Df.res	AUC	BIC	AICc	ΔAICc	*p* value
**f(Area + Genus + Trap)**^a^	**9**	**97**	**0.87**	**137.9**	**113.6**	**0**	**< 0.0001**
**f(Area + Species + Trap)**	**12**	**94**	**0.88**	**146.2**	**115.5**	**1.9**	**< 0.0001**
f(Area + Genus + Ecomorphological + Trap + Capture)	11	95	0.88	146.8	118.2	4.6	**< 0.0001**
f(Area + Genus + Capture)	7	99	0.83	140.7	120.9	7.3	**< 0.0001**
f(Area + Species + Capture)	10	96	0.85	149.1	122.7	9.1	**< 0.0001**
f(Species + Genus + Ecomorphological + Trap)	11	95	0.88	157.3	122.7	9.1	**< 0.0001**
f(Area + Genus + Ecomorphological + Capture)	8	98	0.83	145	122.9	9.3	**< 0.0001**
f(Species)	7	99	0.77	150.5	130.6	17	**< 0.0001**
f(Area + Ecomorphological + Capture)	5	101	0.75	150.1	135	21.4	**< 0.0001**
f(Area + Ecomorphological + Capture)	5	101	0.75	150.1	135	21.4	**< 0.0001**
f(Species + Genus)	7	99	0.77	164.4	138	24.4	**< 0.0001**
f(Capture)	2	104	0.67	146.4	138.6	25	0.0002
f(Species + Genus + Ecomorphological)	8	98	0.77	168.1	139.5	25.9	**< 0.0001**
f(Area + Capture)	4	102	0.70	153	140.3	26.7	0.0007
f(Genus:Area)^b^	8	98	0.77	178.4	145.7	32.1	**< 0.0001**
f(Area + Ecomorphological)	4	102	0.66	158.7	146	32.4	0.0096
f(Area)	3	103	0.56	161.3	151.1	37.5	0.1495
f(Species + Genus + Genus:Area)	11	95	0.79	197.3	155.6	42	**< 0.0001**
f(Species:Area)	13	93	0.79	215.4	167.6	54	**< 0.0001**
f(1)	1	105	0.5	154.4	149.2	35.6	Null

Models are ranked based on ΔAICc, and models with values < 2 are considered equally valid (bold). All variables are explained in the text.

^a^A nomenclature specification of the form f(first + second) indicates all the terms in first together with all the terms in second with any duplicates removed (i.e. additive effects of factors).

^b^A specification of the form f(first:second) indicates the set of terms obtained by taking the interactions of all terms in first with all terms in second.

**Table 3:** Comparison of generalized linear models used to describe cortisol levels in all small mammal species in forest fragment samples.

Cortisol logistic models	Rank	Df.res	AUC	BIC	AICc	ΔAICc	*p* value
**f(Area + Genus + Trap)**	**9**	**97**	**0.94**	**102.5**	**78.2**	**0.0**	**< 0.0001**
**f(Area + Genus + Capture)**	**7**	**99**	**0.92**	**99.65**	**79.8**	**1.7**	**< 0.0001**
f(Area + Species + Trap)	12	94	0.95	111	80.4	2.2	**< 0.0001**
f(Area + Species + Capture)	10	96	0.94	108.2	81.7	3.6	**< 0.0001**
f(Area + Genus + Ecomorphological + Capture)	8	98	0.92	104.3	82.2	4.1	**< 0.0001**
f(Area + Genus + Ecomorphological + Trap + Capture)	11	95	0.94	111.7	83.1	4.9	**< 0.0001**
f(Area + Ecomorphological + Capture)	5	101	0.89	101.3	86.1	8.0	**< 0.0001**
f(Area + Ecomorphological + Capture)	5	101	0.89	101.3	86.1	8.0	**< 0.0001**
f(Species + Genus + Ecomorphological + Trap)	11	95	0.93	129.1	94.5	16.3	**< 0.0001**
f(Species + Genus + Ecomorphological + Trap + Capture)	12	94	0.93	133.7	97.2	19.0	**< 0.0001**
f(Species)	7	99	0.82	122.3	102.5	24.3	**< 0.0001**
f(Area + Ecomorphological)	4	102	0.77	117.2	104.5	26.3	**< 0.0001**
f(Species + Genus)	7	99	0.82	136.3	109.8	31.6	**< 0.0001**
f(Species + Genus + Ecomorphological)	8	98	0.82	141	112.4	34.2	**< 0.0001**
f(Area + Capture)	4	102	0.78	126.9	114.2	36.0	**< 0.0001**
f(Genus:Area)	8	98	0.83	148.5	115.8	37.6	**< 0.0001**
f(Capture)	2	104	0.72	128.8	121.1	42.9	**< 0.0001**
f(Species + Genus + Genus:Area)	11	95	0.85	168.2	126.5	48.3	**< 0.0001**
f(Area)	3	103	0.62	139.8	129.5	51.3	0.0013
f(1)	1	105	0.50	142.3	137.1	58.9	Null

Models are ranked based on ΔAICc, and models with values < 2 are considered equally valid (bold). All variables are explained in text.

To confirm that the trap method did not impact our findings relating to the size of the forest fragment and taxonomic group, we performed binomial models on the data sets, excluding data from individuals trapped via Sherman traps. The results were congruent with the results of the models of the data set that includes the Sherman traps (Supplementary Table 1).

### Glucocorticoid post-hoc results

Because 91.5% of the samples represented individuals in one of two genera (*Oligoryzomys* or *Akodon*), we present the following results to examine glucocorticoid patterns at smaller scales. Of all four genera, *Oligoryzomys* had the greatest mean corticosterone and cortisol concentrations (Table 1).

For the three species of *Oligoryzomys* (*O*. *flavescence*, *O*. *mattogrossae*, and *O*. *nigripes*), there was no difference in neither corticosterone (H_2_ = 2.56, p = 0.28) nor cortisol levels (H_2_ = 1.89, p = 0.39) among the three species. For the two species of *Akodon* (*A*. *montensis* and *A*. *paranaensis*), there was no difference in either corticosterone (U = 146.0, n = 37, p = 0.64) or cortisol levels (U = 156.0, n = 37, p = 0.88) between the two species. Corticosterone and cortisol levels were correlated when evaluating *Oligoryzomys* (ρ = 0.83; n = 62, p < 0.001) and *Akodon* (Spearman’s rho = 0.86; n = 35, p < 0.001) individually. There was no difference between males and females in their corticosterone (*Oligoryzomys*: U = 413.0, n = 60, p = 0.58; *Akodon*: U = 115.0, n = 33, p = 0.58) or cortisol (*Oligoryzomys*: U = 435.0, N = 60, p = 0.82; *Akodon*: U = 91.0, n = 33, p = 0.15) levels.

**Figure 3:**
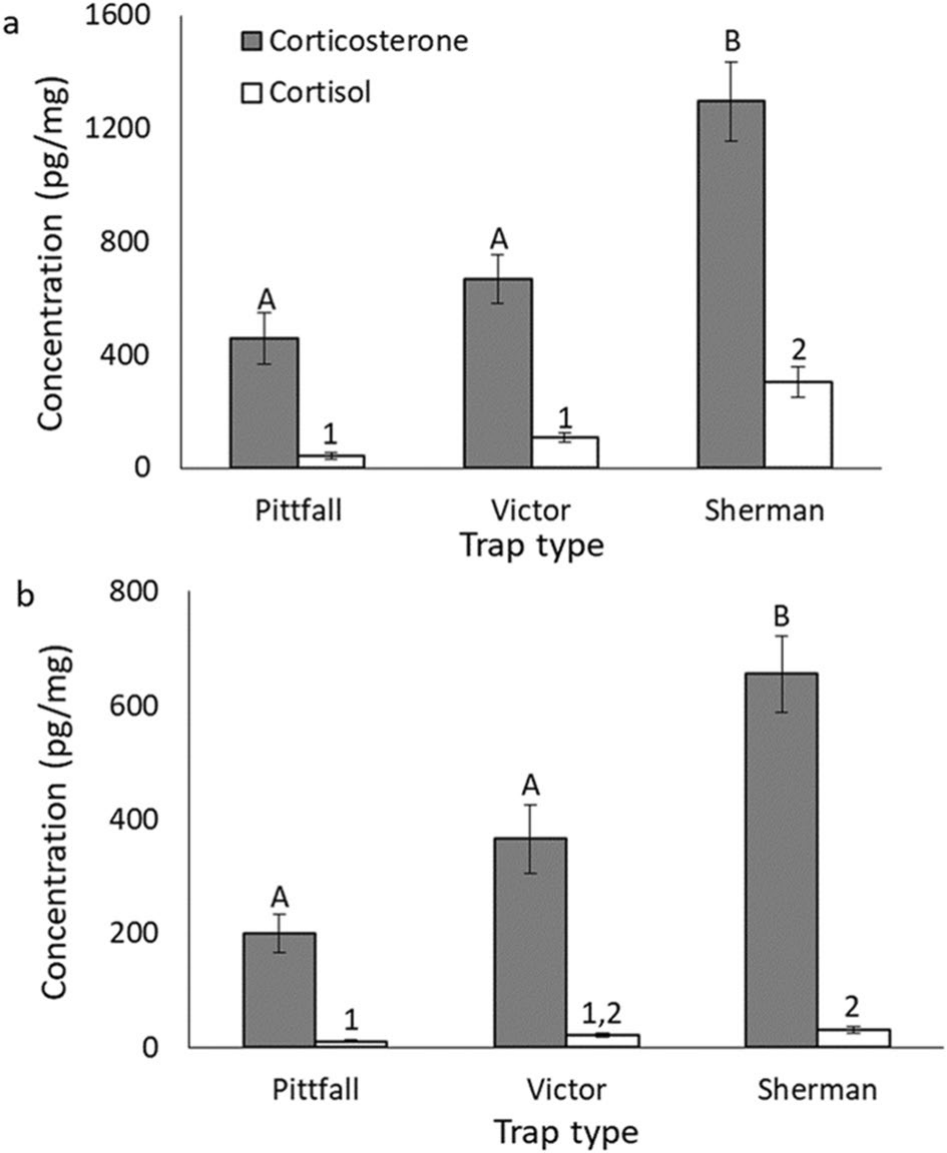
Comparison of glucocorticoid concentration in *Oligoryzomys* (**a**) and *Akodon* (b) samples based on the type of trap (pitfall, Victor, or Sherman) used for sampling the small mammals. Pairwise differences between trap types are indicated by uppercase letters (corticosterone) and numbers (cortisol) above the bars. Although the type of trap may not directly determine glucocorticoid levels per se, glucocorticoids extracted from the fur of the animals may be associated with the trap type (e.g., the amount of time spent in the trap, and if animal was captured alive or died immediately after capture).

For both *Oligoryzomys* and *Akodon* there was a difference in both corticosterone (*Oligoryzomys*: H_2_ = 19.51, p < 0.001; *Akodon*: H_2_ = 11.40, p = 0.003) and cortisol (*Oligoryzomys*: H_2_ = 20.60, p < 0.001; *Akodon*: H_2_ = 7.0, p = 0.030) levels based on the type of trap used; corticosterone and cortisol levels were greatest for individuals captured using Sherman traps (Fig. 3).

## Discussion

We found that forest fragment area, capture mode (type of trap), and taxon were all related to corticosterone and cortisol levels in small mammals. First, as predicted, higher levels of glucocorticoids were more prevalent in individuals captured in the small forest fragments. Second, an unintended but important finding from our study was that the type of trap used for capturing the small mammals impacted the glucocorticoid levels associated with the individual animals; this pattern was corroborated when *Oligoryzomys* and *Akodon* were analyzed separately, too. While trap type does not determine glucocorticoid levels per se, factors correlated with trap type (e.g., if the animal was captured alive or immediately died, length of time alive in the trap) may impact the quantification of the glucocorticoid levels. Finally, as expected, glucocorticoid levels differed greatly among the four genera, and there were specific differences between the rodents and marsupials.

Our first finding, that glucocorticoids were overall higher in individuals living in the small forest fragments, was similar to previous studies that found that primates living in forest fragments had higher levels of glucocorticoids than individuals living in continuous forest habitat^36,39,40^. In our study, individuals in the largest fragment (1200 ha) had glucocorticoid levels that were a fraction of the levels found in individuals in the smaller fragments. Therefore, the individuals in these smaller forest fragments appear to be experiencing increased physiological stress. Increased stress may impact animal health, fitness, the conservation of populations, and emerging infectious diseases^16,46,55^. That said, the patterns are not fully evident across taxa so conclusions should not be painted broadly without further study^34,56^. Although our findings support our hypothesis that forest fragmentation impacts glucocorticoid levels in small mammals, more sampling is needed, given that we sampled only six forest fragments, and two-thirds of the forest fragments in the current study were 25 ha and smaller. In order to draw stronger conclusions on relationships between fragment size and glucocorticoid levels, we would want to expand our sampling of larger fragments (1000 ha and larger).

Our second finding was that the type of trap used to capture the individuals impacted the glucocorticoid levels noted for the individual animals. Typically, it is thought that the use of fur is appropriate for longer-term (days to months) quantification of mean hormone levels^21^. However, we found that individuals trapped in Sherman traps, which typically resulted in live captures and the animals spending hours in the trap post capture, had elevated levels of both corticosterone and cortisol. Although the fur was rinsed with isopropanol prior to hormone extraction, it appears that hormones were absorbed into the hair shaft between the time the animal was captured and when the hormone extraction was completed months later. Hair can absorb water and its volume changes with relative humidity^57^; furthermore, hair lipids including cholesterol are lost when hair is washed and many of these lipids are then replaced by sebum secretion onto the hair shaft^58^. It therefore makes sense that steroid hormones, which all derive from cholesterol, can potentially also enter fur from urine. Therefore, traps that constrain a live animal for a duration of minutes to hours allow time for urine, which shows a faster response to glucocorticoid changes, to potentially be excreted and impact fur glucocorticoid concentrations. Our findings illustrate the important ramifications that sampling type may have on glucocorticoid quantification, even when working with fur. Importantly, our finding of higher glucocorticoid levels in animals from smaller fragments was robust when controlling for the trap type employed.

Our third finding, that glucocorticoid levels differed by mammal taxa, is very important for understanding patterns in glucocorticoid levels across taxonomic groups. The stress response, measured by glucocorticoid levels, varies among and within taxa^17,59^. Our study is the first to present glucocorticoid levels in free-ranging individuals representing these four mammalian genera. These genera represent mammalian lineages that diverged approximately 200 mya, and represent different evolutionary histories in South America^60^. We found that overall, the two rodent genera exhibited high total glucocorticoid levels with corticosterone levels many-fold higher than cortisol levels. In contrast, the two marsupial genera exhibited relatively low total glucocorticoid levels, and their cortisol and corticosterone levels were relatively similar. We found large ranges between minimum and maximum glucocorticoid levels within a genus, but no differences among species within a genus, suggesting that anthropogenic changes may be driving the variance seen across individuals. These results highlight the importance of assaying and analyzing glucocorticoids differently between rodent and marsupial taxa. Different analyses could be especially important when the findings are used for assessing stress in conservation contexts.

Our study greatly adds to the literature on glucocorticoid concentrations in wild small mammals, specifically animals in the genera *Oligoryzomys*, *Akodon*, *Gracilinanus*, and *Marmosa*. In a review of the published literature, we found only one publication on *Gracilinanus* that provided information regarding glucocorticoid concentrations in these small mammal genera^54^. Our study provides fur corticosterone and cortisol levels for free-ranging small mammals in a fragmented landscape, and emphasizes the need for a better understanding of these glucocorticoid levels for animals living in less-disturbed forest environments. When examining glucocorticoid responses, variations often exist between species, reproductive state, time of day, sex, environmental conditions, and methodologies for collecting and analyzing the samples^61^. There is variation in individual glucocorticoid levels, and such variation is important to consider when comparing only population means^27^.

Across animal taxa, the impacts of stress on glucocorticoid levels have been noted, but the extent to which these physiological changes impact the health, survival, and conservation of populations is not fully clear^34,56^. Furthermore, we highlight the important implications that trapping method may have on glucocorticoid measurements, even when one is using fur for measures of longer-term profiles.

## Conclusions

We found that the levels of the glucocorticoids cortisol and corticosterone differed in small mammals based on (1) the size of the forest fragment where the individuals lived; (2) the trapping method used, probably due to stress of confinement upon capture, and absorption of hormones prior to extraction and analysis; and (3) taxon. Our findings suggest that individuals living in heavily disturbed habitats may experience more physiological challenges than individuals in more intact habitats that it is important to take trapping method (trap type) into consideration when analyzing glucocorticoids from fur, and that South American Interior Atlantic Forest rodents and marsupials differ markedly in their glucocorticoid levels.

## Methods

### Field data collection

**Figure 4:**
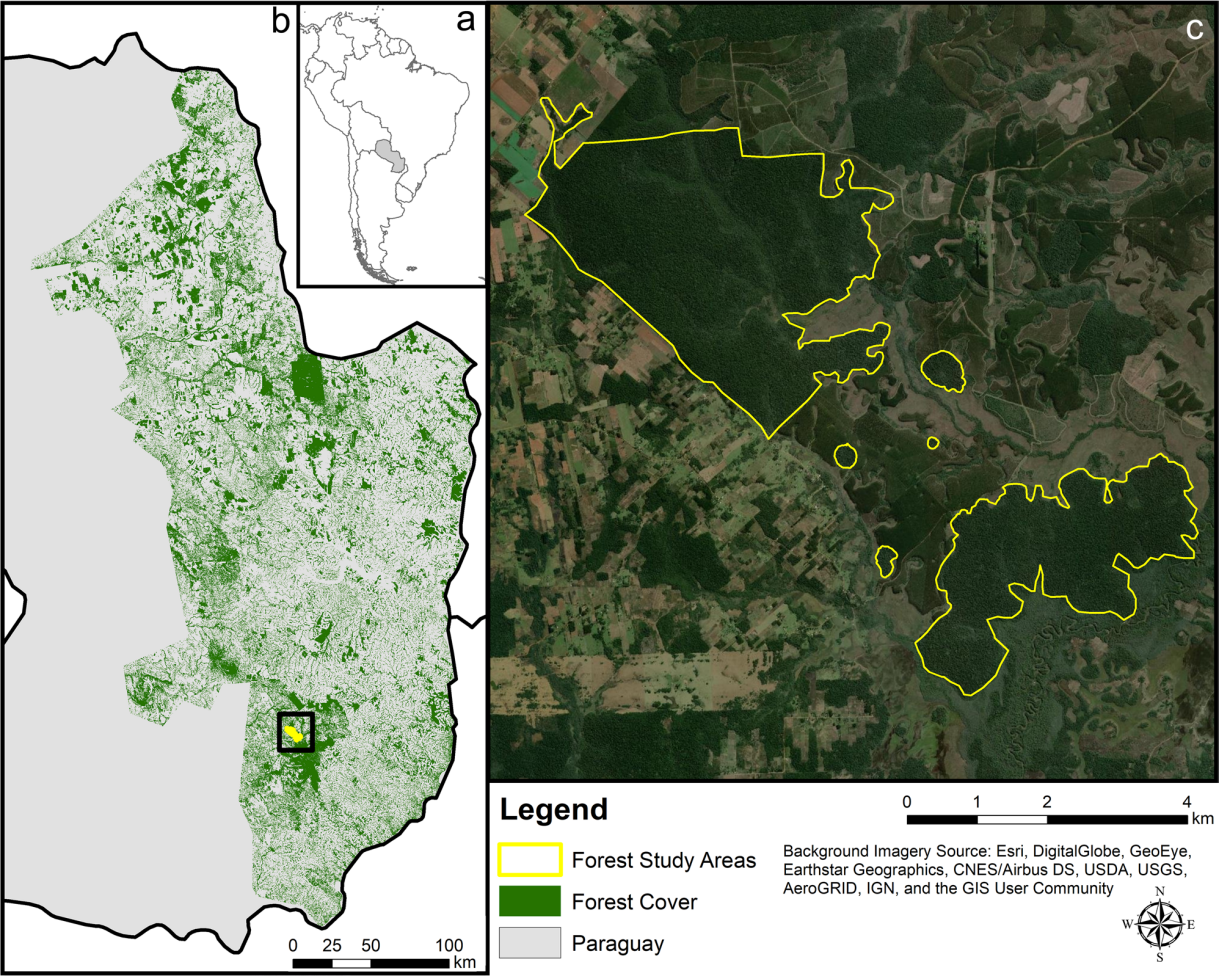
The study area in (**a**) Paraguay at the (**b**) Reserva Natural Tapytá, in the Caazapá Department of Paraguay is located within the highly fragmented Interior Atlantic Forest. The (**c**) six forest fragments in the current study were surrounded by a matrix of gallery forest, wetlands, pasture, and eucalyptus plantations. Small mammals in the four smaller forest fragments (2–25 ha) had higher glucocorticoid levels than small mammals in the larger forest fragments (633 and 1200 ha). Data for forest cover were derived from Hansen et al.^48^ Maps generated using ArcGIS 10.7 (https://desktop.arcgis.com)^91^.

The study was conducted at the Reserva Natural Tapytá, located in the Caazapá Department, Paraguay. The 4736-ha reserve consists of a mosaic of Interior Atlantic Forest, gallery forest, wetlands, pasture, and eucalyptus plantations^62^. The eucalyptus plantations were actively harvested during the study period, but the distance between these harvested areas and the forest fragments we sampled was > 2 km. We collected data in six forest fragments (Fig. 4) that varied in size from 2 to 1200 ha (2, 8, 9, 25, 633, and 1200 ha). Sites were separated from each other by 0.5–2.4 km, with the minimum distance between two forest fragments ranging from 0.5 to 0.8 km (mean ± SE: 0.6 ± 0.04 km). The matrix surrounding the forest fragments primarily consisted of wetlands and pasture. We selected the sample sites based on their availability within the reserve: at the time of sampling the four smaller fragments were fully isolated fragments that were not near harvesting activity in the eucalyptus plantations. There were only two larger forest fragments within this area of the reserve, and we sampled both. Several of the smaller forest fragments showed signs that cattle regularly entered the area. There was no fencing to prevent cattle from entering any of the forest fragments in the reserve. This research followed the American Society of Mammalogists’ guidelines^63^ and was approved by the Institutional Animal Care and Use Committee (IACUC) at Rhodes College, Memphis, Tennessee.

In each of the six forest fragments, we set up one trapping grid. Grids consisted of four parallel traplines, with a pitfall line along the middle of the four main traplines for a total of five lines. Each trapline and pitfall was 10 m apart. Each trapline included 15 stations each approximately 8 m apart. We opted for this approach for comparative purposes with prior sampling conducted in larger forest fragments in the region, and because this approach has been shown to be effective in this habitat^64^. However, we scaled the grids back proportionally in size because the grid size in the earlier studies of the large forest fragments was larger than some of the forest fragments in the current study. Each station along the four traplines consisted of two Sherman traps and two Victor snap traps, with both trap types set on the ground and 1.5 m above the ground on a tree limb. The pitfall line in the middle of the grid had seven pitfall buckets along the line. We sampled two separate fragments concurrently, with sampling occurring in July and August 2013, which coincided with the dry season for the region. In each fragment, we sampled for 8 consecutive nights, and we checked the traps every morning. All grids were placed 5–10 m from the closest edge of the forest fragment.

We collected each individual captured as a voucher specimen, and we documented each individual’s genus, sex, and age; recorded external body measurements; and cut a fur sample (close to the skin but avoiding living follicles) from the lower dorsal region, along the lateral portion of the hind leg. We identified species using craniodental characters, combined with DNA barcoding of selected specimens, which we have found to effectively discriminate among rodent and marsupial species in the region^65–68^. All specimens are currently housed at the Field Museum of Natural History, Chicago, Illinois, USA (Supplementary Table 2).

Measuring glucocorticoid concentrations from hair has shown to work in a number of mammalian species: chimpanzee, *Pan troglodytes*^36^; grizzly bear, *Ursus arctos*^69^; rhesus macaque, *Macaca mulatta*^70^; rock hyrax, *Procavia capensis*^71^. Fur samples were weighed on a precision balance (Denver Instruments Company XE Series model 100a) and these samples ranged from 2 to 19.5 mg for each small mammal sampled, and the entirety of the sample was used in the analysis. There was no difference among the mammal genera in the amount of fur sampled (ANOVA: *F*_102,3_ = 1.25, *P* = 0.30). To test if low fur weights were correlated to low hormone concentrations, we performed a Pearson one-sided permutation correlation test (H_0_: r ≤ 0, H_a_: r > 0)^72^. We found no statistical support for a positive correlation between fur weight and hormone concentration, both for corticosterone (r = − 0.34, p_(r ≤ 0)_ > 0.05, N = 106) and for cortisol (r = − 0.19, p_(r ≤ 0)_ > 0.05, N = 106). Fur samples were stored in Eppendorf tubes until analysis.

### Glucocorticoid assays

We performed steroid analyses of fur samples according to standard protocols^73^. We rinsed the fur twice with isopropanol to remove surface-level steroids, such as those found in sweat. Once the fur had dried, we homogenized the fur by bead beating. Steroid hormones were extracted using 1.5 ml methanol for 24 h. After centrifugation, we allocated 0.5 ml of the supernatant for the corticosterone assay and another 0.5 ml for the cortisol assay. We dried down the methanol using a speed vac, and then reconstituted the samples with the respective enzyme immunoassay kit buffers. We conducted the enzyme immunoassays according to the product directions (Arbor Assays corticosterone kit K014 and cortisol kit K003). We reran samples whose values were above the maximum detectability at 20% of original concentration, along with a subset of original samples. Diluted samples showed parallelism (coefficient of variations: 14.6% for corticosterone, 20.7% for cortisol), and diluted samples correlated strongly with undiluted samples (corticosterone: *r* = 0.86, *n* = 16, *P* < 0.001, cortisol: *r* = 0.99, *n* = 31, *P* < 0.001). The mean intra-assay coefficient of variation for corticosterone was 8.3% and for cortisol it was 13.8%. The inter-assay coefficient of variation for corticosterone was 5.5% and for cortisol it was 19.5%.

### Discriminant function analyses

We used discriminant function analyses (DFA) to optimize the difference between rodent (*Akodon* and *Oligoryzomys*) and marsupial (*Marmosa* and *Gracilinanus*) genera based on corticosterone, cortisol, and the ratio of corticosterone to cortisol using log-transformed matrices. Such analyses aimed to maximize separation between groups based on eigenvectors that represent variables that explain said groups^74^. This was followed up with a non-parametric multivariate analysis of variance (MANOVA) with 10,000 permutations using Matlab function “Dfa” using functions created using a Matlab script created by R. E. Strauss (http://www.faculty.biol.ttu.edu/Strauss/Matlab/Matlab.htm), using log-transformed matrices (see the applications by Hernandez et al.^75^ and Rossi et al.^76^). Taking the non-parametric approach was valuable because it mitigated the conventional assumptions of multivariate normality and homogeneity of covariance matrices^77^ and potentially low sample size. This approach is different from a PerMANOVA sensu Anderson^78^. MANOVA statistical significance was evaluated on a α = 0.05.

### Generalized linear models

To determine the factors or combinations of factors that explained cortisol and corticosterone levels in our samples, we used generalized linear models (GLM). We conducted separate models for cortisol and corticosterone. GLMs are valuable because they allow for the implementation of both categorical and/or continuous explanatory variables^79^. We implemented a binomial family with a logistic link function to account for proportion data based on eight categorical variables: genus, species, ecomorphological (arboreal vs. cursorial), trap type (small Victor, large Victor, pitfall, or Sherman), capture (dead vs. alive immediately prior to fur sampling), trap placement (ground, pitfall, or elevated), and forest fragment size (classified fragment size into small: ≤ 25 ha; medium: 633 ha; and large: 1200 ha).

We implemented an a priori mean-based discretization-threshold approach to discretize the levels of each hormone as the GLM response variable, such that ''high'' indicated the cases that were above the mean of all samples and ''low'' indicated cases that were equal or smaller than the mean. Discretization is a useful preprocessing technique in many knowledge discovery and data mining tasks^80,81^. The induction tasks can benefit from discretization: rules with discrete values are normally shorter and more understandable, discretization can lead to improved predictive accuracy and the discretization can provide nonlinear relations^81^. In this sense, although there are methods of data science that automate discretization, we used mean-based discretization threshold because it is a simple and reproducible criterion.

We conducted these analyses using the function ‘glm’ in the package stats in R v3.6.2^82^. We tested the model statistical significance (α = 0.05) via a likelihood-ratio test with respect to a null model; this approach permitted us to do a statistical test of the goodness-of-fit between two models. We compared each relatively more-complex model with the null model to test that these models significantly outperformed random models. We proceeded to compare the top models based on second-order bias-corrected Akaike Information Criterion AICc^83^. This approach has a larger penalty term than AIC particularly when *n* (the sample size) is small with respect to the number of estimated parameters *k*^84^. The best model was that which had the lowest AICc value. AIC and Bayesian Inferential Criterion^85^ values were also compared using the function ‘compareGLM’ in package rcompanion^86^ in R.

In addition, we computed the area under the receiver operating characteristics curve (AUC) to measure the performance of the models. This approach allowed us to determine two aspects of the models: first, if the model made the predictions randomly (AUC ≈ 0.5); and second, how well the model performed the binary classification task—when the AUC is closer to 1 the performance of the model is better^87,88^. We implemented mosaic plots to illustrate how each hormone level (high or low) was associated with each variable in the best-fit models. Mosaic plots allow the display of values in contingency tables which are cross-classified by one or more factors, where the area in the plots is divided into bars representing quantities of variables that are compared^89^. This pattern of association was statistical followed up with G-tests^90^ of independence with Williams' correction for the small sample size (function GTest form library DescTools in R).

### Analyses by genera

Because 91.5% of the individuals sampled represented two genera, and because this study is the first to report glucocorticoid levels for free-ranging individuals from these mammalian genera, we followed the above analyses with post hoc analyses to examine genus-specific patterns in glucocorticoids in more detail.

For each of the two genera, we tested if there were differences between the species in a particular genus using a non-parametric Mann–Whitney U test when there were two species and non-parametric Kruskal–Wallis test when there were three species. Within each genus, we tested if there was a correlation between corticosterone and cortisol levels using Spearman’s rank correlation, and then tested if there were sex differences in corticosterone and cortisol levels using Mann Whitney tests. We conducted further analyses on the influence of trap type on glucocorticoid levels by using a Kruskal–Wallis test for each genus individually. We used nonparametric tests for these analyses because these tests are insensitive to serial correlation, non-normality, and outliers compared to parametric approaches^79^. All analyses were performed using α = 0.05.

## Supplementary Information

Supplementary Figure 1.

Supplementary Information.
