# Supplementary figures and images for: Small mammal glucocorticoid concentrations vary with forest fragment size, trap type, and mammal taxa in the Interior Atlantic Forest
Source: Sci Rep. 2021 Feb 4;11:2111. doi: 10.1038/s41598-021-81073-2 (PMC7862606; doi:10.1038/s41598-021-81073-2)

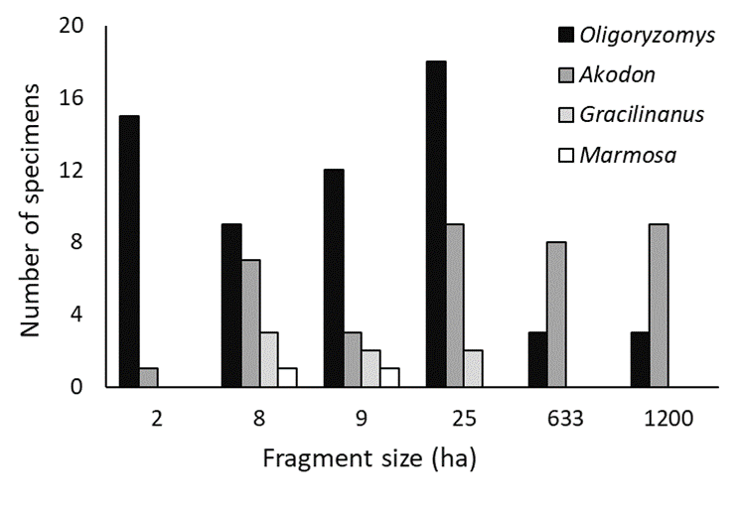

Supplement: Supplementary file 1 — Supplementary Figure 1. [file 41598_2021_81073_MOESM1_ESM.tif]
