# Supplementary material for: Small mammal glucocorticoid concentrations vary with forest fragment size, trap type, and mammal taxa in the Interior Atlantic Forest
Source: Sci Rep. 2021 Feb 4;11:2111. doi: 10.1038/s41598-021-81073-2 (PMC7862606; doi:10.1038/s41598-021-81073-2)
Supplement: Supplementary file 2 — Supplementary Information. [file 41598_2021_81073_MOESM2_ESM.docx]

**Small mammal glucocorticoid concentrations vary with forest fragment size, trap type, and mammal taxa in the Interior Atlantic Forest**

Sarah A. Boyle^1*^, Noé U. de la Sancha^2,3^, Pastor Pérez^4^, David Kabelik^5^

^1^Rhodes College, Department of Biology and Program in Environmental Studies and Sciences, Memphis, Tennessee, USA; sarahannboyle@gmail.com

^2^Chicago State University, Department of Biological Sciences, and ^3^The Field Museum, Integrative Research Center, Chicago, Illinois, USA; delasancha@msn.com

^4^Facultad Politécnica, Universidad Nacional de Asunción, Asunción, Paraguay peperez.estigarribia@gmail.com

^5^Rhodes College, Department of Biology and Program in Neuroscience, Memphis, Tennessee, USA; kabelikd@rhodes.edu

*Corresponding author: sarahannboyle@gmail.com

Supplementary Figure 1. Distribution of four mammal genera captured and assayed for glucocorticoids, across the six forest fragments.

Supplementary Table 1. Model results when data from Sherman traps were excluded from analysis for corticosterone and cortisol. Models are ranked based on ΔAICc, and models with values < 1 are considered equally valid (bold). All variables are explained in text.

| Corticosterone logistic models | Rank | Df.res | AUC | BIC | AICc | ΔAICc | *p* value |
| --- | --- | --- | --- | --- | --- | --- | --- |
| **f(Species)** | **5** | **48** | **0.7558** | **69.86** | **59.86** | **0** | **0.004848** |
| **f(FS+Genus+Trap)** | **7** | **46** | **0.85** | **72.36** | **59.87** | **0.01** | **0.002093** |
| f(FS+Species+Trap) | 9 | 44 | 0.8481 | 75.87 | 61.41 | 1.55 | 0.001342 |
| f(FS+Species+Capture) | 8 | 45 | 0.8077 | 75.51 | 61.96 | 2.1 | 0.002828 |
| f(FS+Genus+Capture) | 6 | 47 | 0.8 | 73.34 | 62.04 | 2.18 | 0.00772 |
| f(FS+Genus+Ecomorphological+Trap+Capture) | 9 | 44 | 0.8692 | 77.68 | 63.21 | 3.35 | 0.002585 |
| f(1) | 1 | 52 | 0.5 | 66.99 | 63.29 | 3.43 | null |
| f(FS+Ecomorphological) | 4 | 49 | 0.7115 | 72.12 | 63.55 | 3.69 | 0.035 |
| f(Capture) | 2 | 51 | 0.5375 | 69.22 | 63.8 | 3.94 | 0.1261 |
| f(FS) | 3 | 50 | 0.5981 | 71.14 | 64.09 | 4.23 | 0.07504 |
| f(FS+Genus+Ecomorphological+Capture) | 7 | 46 | 0.8038 | 76.91 | 64.43 | 4.57 | 0.01158 |
| f(FS+Ecomorphological+Capture) | 5 | 48 | 0.7346 | 74.55 | 64.55 | 4.69 | 0.03238 |
| f(FS+Ecomorphological+Capture) | 5 | 48 | 0.7346 | 74.55 | 64.55 | 4.69 | 0.03238 |
| f(FS+Capture) | 4 | 49 | 0.6173 | 73.85 | 65.27 | 5.41 | 0.07166 |
| f(Species+Genus) | 5 | 48 | 0.7558 | 77.8 | 65.31 | 5.45 | 0.004848 |
| f(Species+Genus+Ecomorphological) | 6 | 47 | 0.7558 | 81.77 | 68.22 | 8.36 | 0.009304 |
| f(Species+Genus+Ecomorphological+Trap) | 8 | 45 | 0.8173 | 83.85 | 68.62 | 8.76 | 0.003281 |
| f(Genus:FS) | 7 | 46 | 0.8 | 84.23 | 69 | 9.14 | 0.002063 |
| f(Species+Genus+Genus:FS) | 9 | 44 | 0.85 | 90.85 | 74.32 | 14.46 | 0.0009584 |
| f(Species:FS) | 11 | 42 | 0.85 | 102.8 | 86.75 | 26.89 | 0.00285 |
|  |  |  |  |  |  |  |  |
| Cortisol Logistic Models | Rank | Df.res | AUC | BIC | AICc | ΔAICc | *p* value |
| **f(FS+Genus+Trap)** | **7** | **46** | **0.90** | **56.75** | **44.26** | **0** | **0.0011** |
| **f(FS+Ecomorphological)** | **4** | **49** | **0.78** | **53.69** | **45.12** | **0.86** | **0.0051** |
| f(Species) | 5 | 48 | 0.79 | 56.64 | 46.64 | 2.38 | 0.0069 |
| f(FS+Genus+Capture) | 6 | 47 | 0.85 | 57.97 | 46.66 | 2.4 | 0.0046 |
| f(FS+Ecomorphological+Capture) | 5 | 48 | 0.81 | 56.69 | 46.69 | 2.43 | 0.0071 |
| f(FS+Ecomorphological+Capture) | 5 | 48 | 0.81 | 56.69 | 46.69 | 2.43 | 0.0071 |
| f(1) | 1 | 52 | 0.50 | 52.92 | 49.22 | 4.96 | null |
| f(FS+Genus+Ecomorphological+Capture) | 7 | 46 | 0.85 | 61.94 | 49.45 | 5.19 | 0.0083 |
| f(FS+Genus+Ecomorphological+Trap+Capture) | 9 | 44 | 0.90 | 64.54 | 50.08 | 5.82 | 0.0036 |
| f(FS+Species+Trap) | 9 | 44 | 0.90 | 64.69 | 50.23 | 5.97 | 0.0038 |
| f(Capture) | 2 | 51 | 0.54 | 56.23 | 50.81 | 6.55 | 0.3509 |
| f(FS) | 3 | 50 | 0.60 | 58.56 | 51.52 | 7.26 | 0.1585 |
| f(FS+Species+Capture) | 8 | 45 | 0.85 | 65.38 | 51.83 | 7.57 | 0.0115 |
| f(Species+Genus) | 5 | 48 | 0.79 | 64.58 | 52.09 | 7.83 | 0.0069 |
| f(FS+Capture) | 4 | 49 | 0.65 | 61.17 | 52.6 | 8.34 | 0.1224 |
| f(Species+Genus+Ecomorphological+Trap) | 8 | 45 | 0.88 | 69.54 | 54.3 | 10.04 | 0.003 |
| f(Species+Genus+Ecomorphological) | 6 | 47 | 0.79 | 68.55 | 55 | 10.74 | 0.0129 |
| f(Species+Genus+Ecomorphological+Trap+Capture) | 9 | 44 | 0.88 | 73.45 | 57.61 | 13.35 | 0.005 |
| f(Genus:FS) | 7 | 46 | 0.83 | 74.48 | 59.25 | 14.99 | 0.0105 |
| f(Species+Genus+Genus:FS) | 9 | 44 | 0.84 | 85.81 | 69.3 | 25.0 | 0.0209 |

Supplementary Table 2. List of voucher specimens, including museum catalog numbers and field collection number in parenthesis, which are housed at the Field Museum of Natural History in Chicago, Illinois, USA.

| Order | Family | Genus | Species | Voucher numbers |
| --- | --- | --- | --- | --- |
| Rodentia | Cricetidae | *Akodon* | *montensis* (n=23) | 232559 (ND-660), 232548 (ND-663), 232549 (ND-664), 232550 (ND-665), 232558 (ND-669), 232560 (ND-670), 232561 (ND-671), 232562 (ND-672), 232551 (ND-674), 232552 (ND-678), 232553 (ND-681), 232572 (ND-684), 232573 (ND-689), 232594 (ND-690), 232595 (ND-695), 232596 (ND-698), 232597 (ND-700), 232574 (ND-704), 232575 (ND-719), 232600 (ND-723), 232577 (ND-727), 232601 (ND-733), 232602 (ND-734) |
| Rodentia | Cricetidae | *Akodon* | *paranaensis* (n=14) | 232565 (ND-667), 232547 (ND-673), 232563 (ND-677), 232566 (ND-680), 232554 (ND-682), 232564 (ND-683), 232578 (ND-692), 232579 (ND-703), 232598 (ND-711), 232599 (ND-713), 232650 (ND-744), 232652 (ND-755), 232651 (ND-758), 232624 (ND-761) |
| Rodentia | Cricetidae | *Oligoryzomys* | *flavescence* (n=2) | 232606 (ND-701), 232626 (ND-757) |
| Rodentia | Cricetidae | *Oligoryzomys* | *mattogrossae* (n=2) | 232657 (ND-751), 232648 (ND-773) |
| Rodentia | Cricetidae | *Oligoryzomys* | *nigripes* (n=56) | 232555 (ND-661), 232556 (ND-662), 232557 (ND-666), 232567 (ND-668), 232568 (ND-675), 232569 (ND-679), 232607 (ND-685), 232608 (ND-686), 232584 (ND-688), 232585 (ND-691), 232586 (ND-693), 232609 (ND-694), 232610 (ND-696), 232611 (ND-697), 232612 (ND-699), 232587 (ND-702), 232588 (ND-705), 232590 (ND-707), 232613 (ND-708), 232614 (ND-709), 232615 (ND-710), 232616 (ND-712), 232591 (ND-714), 232617 (ND-716), 232618 (ND-717), 232570 (ND-722), 232619 (ND-728), 232593 (ND-730), 232620 (ND-732), 232621 (ND-735), 232622 (ND-736), n/a^1^ (ND-737), 232627 (ND-740), 232628 (ND-741), 232629 (ND-742), 232632 (ND-749), 232633 (ND-750), 232637 (ND-756), 232659 (ND-759),232638 (ND-763), 232639 (ND-764), 232640 (ND-765), 232641 (ND-766), 232660 (ND-767), 232643 (ND-769), 232644 (ND-770), 232645 (ND-771), 232646 (ND-772), 232661 (ND-774), 232662 (ND-775), 232664 (ND-778), 232665 (ND-779), 232667 (ND-782), 232668 (ND-783), 232671 (ND-786), 232672 (ND-788) |
| Didelphimorphia | Didelphidae | *Gracilinanus* | *agilis* (n=7) | 232604 (ND-715), 232580 (ND-724), 232605 (ND-726), 232581 (ND-729), 232582 (ND-731), 232653 (ND-743), 232654 (ND-780) |
| Didelphimorphia | Didelphidae | *Marmosa* | *paraguayana* (n=2) | 232583 (ND-739), 232656 (ND-776) |

^1^This specimen was sampled for fur but was not included as a voucher specimen.
